# Racial disparities in the COVID-19 response affecting the Marshall Islands diaspora, United States of America

**DOI:** 10.2471/BLT.20.277855

**Published:** 2021-06-01

**Authors:** Don E Willis, Pearl A McElfish

**Affiliations:** a College of Medicine, Division of Community Health and Research, University of Arkansas for Medical Sciences, 1125 N. College Ave., Fayetteville, AR 72703, United States of America (USA).; b College of Medicine, University of Arkansas for Medical Sciences, Fayetteville, USA.

Racial disparities in rates of infection, hospitalization and death due to coronavirus disease 2019 (COVID-19) became apparent soon after the severe acute respiratory syndrome coronavirus 2 (SARS-CoV-2) arrived in the United States of America (USA). Nationwide, people of colour are two to three times more likely to die from COVID-19 than white Americans.^1^

Native Hawaiians and Pacific Islanders are also at heightened risk for infection, hospitalization and death from COVID-19.^2^ This disparity is particularly acute for the Marshallese, a group of Pacific Islanders whose largest population in the mainland USA is in North-west Arkansas. While Marshallese account for only 1.5–2.4% of the 509 569 population of the two counties of the state, Benton and Washington, in June 2020, they made up 18.8% of all adult COVID-19 cases (647 Marshallese out of 3436 non-Marshallese population who had COVID-19) and 38.1% (8/21) of deaths.^2^ Even among racial and ethnic minorities, the Marshallese stood out as having much higher odds of death due to COVID-19 (approximately 25 times that of Hispanic residents).^2^ As recently as March 2021, Native Hawaiians and Pacific Islanders living in Arkansas experienced infection and death rates of 26 121 and 436 per 100 000, respectively, compared with 8917 and 178 for white Arkansans.^3^

Racial health disparities are often framed in ways that focus on risk factors, such as underlying health conditions or health behaviours.^4^ The idea that race, rather than racism, is at the root of racial health disparities is implicit in this framing. Less attention is paid to social conditions that place racial minorities at risk of risks.^5^ If an individual has diabetes, they may be at risk for severe COVID-19. However, that risk may be contingent on social conditions that shape exposure to the virus. Limited education and opportunities that place workers in jobs with increased risk of exposure, crowded conditions, unpaid sick leave, and pay too low to feel secure in taking time off to quarantine places workers at risk of risk. Further upstream are policies (or lack thereof), institutional racism and socioeconomic relations that allow for such conditions to exist in the first place and lead to the overrepresentation of people of colour in jobs with high exposure. Such social conditions are fundamental and shape who is at risk of risk because they set the stage for a whole host of risks downstream from them, and because social conditions place individuals at risk regardless of the specific mechanisms that link them to any particular disease.^5^

Reports of racial disparities for COVID-19 have been no exception to the pattern of focusing on individual risk factors rather than social conditions, leading to stigma and victim-blaming.^6^ Reporting racial disparities without adequate context leaves social conditions such as racism unchecked and can also contribute to racism and racist ideologies. Reports of racial disparities with no mention of unequal educational or job opportunities, workplace exposure, historical and ongoing systemic racism or political power are lacking context, and racial stereotypes fill in the blanks of omitted context. Dominant racial ideologies include frames that operate as set paths for interpreting information that are characterized by how predictably they interpret racial phenomena.^7^ In a racialized society, such as the USA, leaving explanations for why racial health disparities exist up to readers’ interpretation all but ensures the propagation of victim-blaming and consolidation of biological racial myths.

## Social conditions 

The story of how Marshallese arrived in North-west Arkansas from the Marshall Islands is critical for the understanding of the social conditions that place them at risk of risks during this pandemic. The country is an archipelago within the region of Micronesia and was previously under the administrative control of the USA. Between 1946 and 1958, the US military used the Marshall Island atolls as a nuclear testing ground for the equivalent of 7200 Hiroshima-sized bombs.

Marshallese living on the test sites were relocated to other atolls, while those living on nearby atolls were not relocated and were exposed to nuclear fallout.^8^ Several of the atolls remain more radioactively contaminated than Chernobyl,^9^ and Runit Dome, a large concrete dome on Enewetak Atoll intended to contain radioactive material left over from the nuclear tests, is leaking into the surrounding waters as the sea rises around it.

After 1986, the Compacts of Free Association allowed Marshallese citizens to freely enter, reside and work in the USA. Since then, the population of Marshallese in North-west Arkansas has increased rapidly. Although Marshallese migrants were eligible for Medicaid – a federal and state programme that helps with health-care costs for low-income individuals –when the agreement was first made, the 1996 Personal Responsibility and Work Opportunity Reconciliation Act made them ineligible;^2^ eligibility was not restored until December of 2020. The Marshallese living in Arkansas experience disproportionate rates of many chronic and infectious diseases. While this epidemiology plays a role in their disproportionate COVID-19 burdens, we must consider the social conditions that place them at higher risk of those proximate causes in the first place – the fundamental causes of disease.^5^

Marshallese informants for the Centers for Disease Control and Prevention report noted that they work in jobs that put them at risk of COVID-19, and that they lack adequate economic and social safety net resources.^2^ More than a quarter of the 647 Marshallese cases reported to work in poultry processing facilities and are considered essential workers. Local needs assessments suggest that approximately half of Marshallese do not have insurance even after the Affordable Care Act was enacted. A lack of insurance, language barriers, lack of familiarity with the health-care system and provider stigma have prevented many Marshallese from seeking health care before the COVID-19 outbreak.^10^ Moreover, Marshallese often wait until a health crisis is perceived before seeking care, a pattern related to cultural understandings of health and health care.^11^ These constraints have not changed since the pandemic began and, by December 2020, interviews with the Marshallese Consulate General and the Arkansas Department of Health revealed that four Marshallese had died in their homes from COVID-19 without seeking care (McElfish P, University of Arkansas for Medical Sciences, personal communication, December 2020).

A focus on socioeconomic factors must also account for the history of deliberate displacement and disenfranchisement, a commonality the Marshallese share with many people of colour. The socioeconomic conditions of the Marshallese are inseparable from the structural racism that set in motion their initial relocation from their atolls, the use of their land and sea for nuclear weapons testing, insufficient responses intended to repair those acts and the legal discrimination (or at best, neglect) that has prevented equitable access to health care for decades.

## Contextualizing racial disparities

Social conditions considered to be fundamental causes of disease have an enduring impact even as new diseases such as COVID-19 emerge and the intervening mechanisms change.^5^ Although SARS-CoV-2 is a novel coronavirus, its disproportionate burden across social hierarchies is all too familiar and has highlighted those pre-existing disparities. The theory of fundamental causes suggests that “[they] can defy efforts to eliminate their [own] effects when attempts to do so focus solely on the mechanisms that happen to link them to disease in a particular situation.”^5^ For example, the COVID-19 vaccines are a medical advancement that can be an intervening mechanism to reduce infection and death; however, without understanding how racism and inequality contribute to unequal access, hesitancy and vaccine uptake, these medical innovations may exacerbate rather than reduce the racial gaps in the risk of contracting or dying from the disease. Moreover, efforts that look to increase vaccination behaviour without understanding racism as a fundamental cause of disease will miss important contexts within which health-care seeking or system avoidance behaviours emerge. Finally, without the added context and understanding of fundamental causes of disease, racial health disparities continue to be framed in ways that view race as biological, or explained with a focus on behavioural differences or other proximate risk factors.^4^^,^^12^

Although COVID-19 has had a clear social patterning, we argue that merely reporting disparate patterns of death and disease may perpetuate racial myths. Here we have considered only one group, which limits the extent to which the specific contexts we have described are relevant to other racial minorities. However, this limitation does not diminish our argument that reporting racial disparities without speaking of the context in which they arise can strengthen the ideology of biological race.^12^ This type of reporting contributes to both the interpersonal and institutional forms of racism which are, in turn, contributors to these racial health disparities. We must acknowledge our own role as researchers in challenging or perpetuating racial myths. Reminding ourselves and readers that racial disparities do not appear out of nowhere, but are linked to historical and ongoing structural racism, is one immediate step we can take in challenging rather than perpetuating racial myths.
